# Supplementary material for: Advanced practice providers versus medical residents as leaders of rapid response teams: A 12-month retrospective analysis
Source: PLoS One. 2022 Aug 23;17(8):e0273197. doi: 10.1371/journal.pone.0273197 (PMC9398002; doi:10.1371/journal.pone.0273197)
Supplement: Attachment — Submitted filename: Rebuttal.docx [file pone.0273197.s002.docx]

**Rebuttal:**

We thank de reviewers for their valuable feedback on the manuscript and we consider the requested changes as very helpful. To address the language and scientific writing we sent the manuscript to a native English scientific writer for improvement.

Comments reviewer #1:

1. I would like to see data regarding the levels of training and experience of the two groups of providers (particularly the non-medical team leaders). When other sites consider rolling-out this staffing model, it will be important for them to understand that the response team is led by people who work full time in the ICU and have done for years. This is not likely to be the same resource available to rural centres, limiting the generalisability of the study, hence the importance of stating the setting of this study. (Also add a generalisability comment in your discussion).

Answer: We added the experience of the APPs to the manuscript. We agree that experience is definitely a factor which is important to consider. What also has to be kept in mind that The APPs in this study also evolved from clinicians with very limited capabilities to valuable clinicians. We think that this reflects a dynamic environment which develops itself over years. The new APPs do not have the experience yet, but tend to learn very fast through guidance form the more experienced APPs. In this way this staffing model could also be applicable to healthcare organizations in rural areas. When deploying more APPs, clinical experience will develop faster and easier. It is probably the initial organizational setup which is most difficult. In addition, we experienced a transfer of nurses to become APPs. These nurses are often a selection which has an urge to develop and explore other professions. Because we offered them the opportunity to become an APP, these people continued to participate in our team.

2. I recommend adding the time frame of your study to the abstract and methods.

Answer: We added the timeframe: the pre-COVID period 2017-2018. We chose this period because we think this does better reflect the ordinary duties of the APP and MR than a period during the COVID pandemic.

3. Data are pleural, this should be changed throughout the manuscript.

Answer: this has been corrected together with the language and style by editing of the manuscript by a native scientific writer.

4.The results should start with a participant flow diagram or statement. It is not clear if all rapid response team calls were included in your study at the moment.

Answer: We added this statement at the beginning of the results section. Additionally, we explained why the number of intubations is less than the total number of patients: not all patients received this treatment. We therefore, did not delete all the text after the tables, because we considered it an explanation for the fact that we were not able to retrieve all data on every parameter.

5. There is a sentence/section referring to the FTE of response by various healthcare workers to calls - this can be deleted, the gross numbers and percentages are enough. I think you are trying to convey levels of experience in these comments, which would be better dealt with another way.

Answer: We deleted this section and made a general remark. We did considered this item worth mentioning because we experience some hesitation by the MR to confront critical situations on the normal wards, where the APP seems to consider these situations a challenge.

6. I recommend light editing throughout the manuscript by a native English speaker - the manuscript is well written but there are minor phrase and spelling issues throughout. eg "de" instead of "the"

Answer: We involved a native scientific writer for editing. We think the language and the manuscript has improved considerably.

7. Table 1 should start with the numbers of patients in each column (n=xx)

Answer: We added the number of patients to table 1.

8. A lot of your narrative paragraphs in your results repeat data in Table 2 and could be deleted.

Answer: we deleted a few repetitive parts of the text (as also mentioned by reviewer 2). We left some of the parts describing the results of table 2 unchanged because of the explanatory additional information in it.

9. Whilst you didn't reach statistical significance, your physicians assistants seem to be slower at initiating procedures in your ICU. This might be worth a comment. Some of the times are an hour or so longer - you seem not to have an adequate sample size if this doesn't reach statistical significance.

Answer: We agree with the reviewer, and described this weakness in the limitation section of the manuscript. The relative small number of calls and the additional smaller number of intubations indicate a too small sample size. We added these parameters however, to explore additional parameter besides the raw patient outcome data which are almost always in line with the quality standards in a adequately organized healthcare organization and they do not often point to the favorability of one profession.

10. if word counts are an issue, almost all sections could be more concise.

Answer: Parts of the text were deleted and the suggestions of the scientific writer made the manuscript more concise.

Comments reviewer #2:

This paper compares rapid responses of teams led by a medical resident and by an advance practice provider. It is an interesting approach to broaden and valide skills of rapid response team leaders, based on the MAELOR tool.

1. It does not however consider the role of the ICU nurse who is also part of the team, nor of the prior experiences of the leaders. These should be mentioned in the limitations. The accompanying nurse can play a major role in ensuring the quality of patient management: it is common for junior doctors to ask experienced nurses for their opinions and support, for example, and not considering them at all in the team's performance seems a bit of an oversight.

Answer: We thank the reviewer for addressing this topic. We also value the important additional support and recommendations of the nurse and therefore, added this to the limitations section. In addition, to the other limitations this is another reason why the measurement of separate professions in a team is difficult. Mostly the entire team performance is measured.

2. Overall, the presented analysis seems appropriate and the results are discussed with pertinence. It is interesting to consider making RRT leader specialists, since medical residents can have varying levels of expertise. It will be however important to ensure that medical residents continue to be exposed to and train for emergencies during their postgraduate training.

Answer: We agree with this comment. This is the reason why we have a mixed team with a limited number of APPs although they are considered experienced and valuable for quality and continuity of critical care medicine.

3. There are minor revisions needed for English (including : line 152, experienceS; line 161, no plural for advice; line 205, what is the word "en" ; line 216, correct continues for continuous; the paragraph 274-279 needs revision: the first sentence has no verb, the third is difficult to understand). The section in lines 263-266 are a repetition of the information in lines 247-250.

Answer: We addressed this remark by consulting a native English speaking scientific writer, who offered suggestions for the improvement of the language and style of the manuscript. We think this led to a considerable improvement and a better readable manuscript.
