# Supplementary material for: Advanced practice providers versus medical residents as leaders of rapid response teams: A 12-month retrospective analysis
Source: PLoS One. 2022 Aug 23;17(8):e0273197. doi: 10.1371/journal.pone.0273197 (PMC9398002; doi:10.1371/journal.pone.0273197)
Supplement: Attachment — Submitted filename: Rebuttal second revision.docx [file pone.0273197.s003.docx]

**Rebuttal second review:**

We thank de reviewers for their latest valuable feedback on the manuscript and we consider all requested changes as very helpful.

Comments reviewer #2:

1. Page 10, line 281: “were” instead of “could be”

Answer: Changed

2. Revise figure 1.

Answer: We want to thank the reviewer for this suggestion. We revised the figure and have improved the contrast between the positive (black, white text) and the negative (grey, black text) outcomes of the MAELOR tool. We also improved the directions of the flows in the flowchart.
